# Machine learning based prediction of suicidal behaviors in patients with perinatal depression: a nationwide register-based cohort study

**DOI:** 10.1192/j.eurpsy.2025.338

**Published:** 2025-08-26

**Authors:** H. Yu, E. Hysaj, E. Bränn, D. Lu

**Affiliations:** 1 Institute of Environmental Medicine, Karolinska Institutet, Stockholm, Sweden

## Abstract

**Introduction:**

Previous results show that women who suffered from perinatal depression (PND) have a higher risk of suicidal behavior. However, clinical tools for predicting suicidal behavior among patients with PND are lacking.

**Objectives:**

To develop a prediction model for suicidal behavior after PND using machine learning.

**Methods:**

Leveraging nationwide Swedish register data, we included 57,848 women with PND diagnosed during 2001-2018 and identified 2,303 events of suicidal behavior up to 5 years after PND diagnosis. Based on our previous association studies, 16 predictors containing information on demographics and pregnancy characteristics were included after multiple imputation. The sample was randomly split into 80% as a training set and 20% for testing. Classification and Regression Tree (CART), Random Forest (RF), Naïve Bayes (NB) and Logistic regression (LR) were used to establish prediction models with area under the curve (AUC) assessed for prediction performance. 10-fold cross-validation was used to evaluate the algorithms on unseen data.

**Results:**

In the prediction models, the CART yielded the best performance for suicidal behavior within 5 years after PND diagnosis (AUC 0.78, 95% CI 0.76-0.81). The population that scored highest (17%) in CART model had 84% risk of suicidal behavior. LR also had a comparable performance (AUC 0.78, 95% CI 0.76-0.81), whereas RF (AUC 0.74, 95% CI 0.72-0.76) and NB (AUC 0.70, 95% CI 0.68-0.72) had relatively poor performance. Notably, suicide history was a main contributor in all four models. Other predictors like household income, gestational age and education level were also important indicators of suicidal behavior risk.

**Conclusions:**

The machine learning models have promising prediction performance for suicidal behavior after PND. Yet, further improvement is needed before clinical implementation.

**Disclosure of Interest:**

None Declared

